# Code of Ethics

**Published:** 1881-06

**Authors:** 


Code of Ethics.—"Rachis calls attention to some of the *uses* made of the Code of Ethics," in another part of this issue, and unless we have been misinformed there are other uses, or rather abuses, of the laws intended for the government of the profession than those to which he refers, and which should be corrected; or abolish the Code altogether, and thus allow the present violators the privilege of the celebrated Kilkenny cats. Gentlemen really do not *need* any such laws as the Code contains to guide *them* in their conduct towards their brethren or the public.

